# The Governance of the Mitigation of the Baltic Sea Eutrophication: Exploring the Challenges of the Formal Governing System

**DOI:** 10.1007/s13280-013-0481-8

**Published:** 2014-01-12

**Authors:** Nina Tynkkynen, Paula Schönach, Mia Pihlajamäki, Dmitry Nechiporuk

**Affiliations:** 1 School of Management, University of Tampere, Tampere, Finland; 2 Department of Environmental Sciences, University of Helsinki, Helsinki, Finland; 3 Environmental Policy Centre, Environmental Governance Studies Unit, Finnish Environment Institute, Helsinki, Finland; 4 Center for Historical Research, Higher School of Economics, National Research University, Moscow, Finland

**Keywords:** Baltic Sea, Environmental policy, Eutrophication, Governance

## Abstract

This article focuses on the governing system of the mitigation of eutrophication in the Baltic Sea. Policies and measures of the Baltic Sea coastal countries, the macro--regional (HELCOM) level, and the level of the European Union are described and governance challenges explicated. We found that the main challenges at different governance levels include: differences between coastal countries in terms of environmental conditions including environmental awareness, overlaps of policies between different levels, the lack of adequate spatial and temporal specification of policies, and the lack of policy integration. To help to meet these challenges, we suggest closer involvement of stakeholders and the public, the improvement of the interplay of institutions, and the introduction of a “primus motor” for the governance of the mitigation of eutrophication in the Baltic Sea.

## Introduction

The Baltic Sea environment is among the most persistently addressed political issues in the Baltic Sea region (e.g., Hjorth 1996; VanDeveer 2011). Various arrangements shape the efforts to protect the Baltic Sea marine environment, including the European Union (EU) and the Helsinki Convention with its governing body the Helsinki Commission, HELCOM. At the same time, the Baltic Sea is regarded as one of the most polluted marine environments in the world. There is a stark contrast between the formally successful governance system and the actual state of the Baltic Sea. In particular, this applies to the problem of eutrophication, which is regarded as the most intricate environmental problem of the Baltic Sea (e.g., Wulff et al. 2007; HELCOM 2009). The extensive nutrient input to the Baltic Sea originates from the large catchment area comprising fourteen countries^1^ with over 85 million people. The main sources of nutrients include natural outflow from land, up-welling of phosphorus-rich deep water (i.e., internal load), atmospheric deposition of nitrogen, and, most importantly, anthropogenic sources such as municipal and single household wastewater, agriculture, and industry (e.g., HELCOM 2009). Eutrophication has negative impacts on recreational activities, biodiversity, and livelihoods, such as fishing and tourism. Some success in the reduction of nutrient load from point sources has been achieved, but nutrient input from diffuse sources, especially from agriculture, is still a major challenge.

The complexity of human activities causing heavy anthropogenic stress, the unique ecological characteristics of the sea, and the dynamic and complex nature of ecological processes make the management of the eutrophication problem intricate. The nature and severity of the problem vary between different basins (see e.g., HELCOM 2009). Nutrient emissions drift from estuaries and coastal waters to the Baltic proper and to the territorial waters of other countries. Russia and Poland are the major sources of waterborne nutrients, but they do not suffer from extensive eutrophication in their territorial waters as their nutrient loads drift to other parts of the Baltic Sea. In terms of societal perceptions of the problem, eutrophication lacks the definitional, ideological, and symbolic clarity typical for many other environmental issues. This makes it speculatively a less “trendy” issue compared with environmental problems such as climate change (Pihlajamäki and Tynkkynen 2011a; see also Lundberg 2013).

In this article, we examine the governing system of the Baltic Sea eutrophication mitigation. According to Kern (2011), the governing system is a social system including different institutions and steering mechanisms that contribute to the development of a “system-to-be governed,” in this case the Baltic Sea ecosystem. The governing system and the “system-to-be-governed” form a dynamic relationship that is affected by the diverse, complex, and vulnerable character of both. The governing system of the Baltic Sea environment originates from the establishment of the Helsinki Convention on the Protection of the Marine Environment of the Baltic Sea area in 1974 (HELCOM 2008; Räsänen and Laakkonen 2008), and has evolved from a single convention to a multifaceted governing system of the common sea especially since the dissolution of the Soviet Union in 1991. The transition of former state socialist countries to market economy, the global agenda on sustainable development, and the enlargement of the European Union, have triggered the development of various cooperative arrangements aiming at managing the Baltic Sea environment over the last three decades (Joas et al. 2008). In this system, international and EU policymaking has gained an increasing importance. At the same time, a variety of new, broader forms of cooperation have emerged including private and public actors (Gänzle 2011; Kern 2011; VanDeveer 2011). Despite the growing number of nongovernmental actors involved, national governance is still considered the backbone of the governing system of the Baltic Sea environment (Kern 2011; Lundberg 2013).

This article portrays a synthesis and describes the main conclusions of the research project PROBALT: “Improving societal conditions for the Baltic Sea protection,” carried out as part of the BONUS+ program during 2009–2011 under the coordination of the Finnish Institute of International Affairs. In the project, main challenges of the formal governing system of the mitigation of the Baltic Sea eutrophication were scrutinized with the help of case studies that focused on the European Union, HELCOM, and all but one of the riparian countries, i.e., Estonia, Finland, Germany, Latvia, Lithuania, Poland, Russia, and Sweden^2^ (for case studies see Pihlajamäki and Tynkkynen 2011b). In this article, we refer to the results of the cases conducted by the authors of this article (Sweden, Finland, Russia, and HELCOM), and on the synthesis articles of the remaining cases, published in the final report of the project by those project members that are not among the authors of this article (Dmochowska and Szaniawska 2011; Hautakangas and Ollikainen 2011; Jokela 2011; Schumacher 2011a, b). While appreciating the growing involvement of a variety of other actors, this article focuses on the formal governing system as set out in the scope of the PROBALT project that is based on the state-centricity of the contemporary governing system (see Kern 2011).

The research data for all case studies consist of policy documents and interviews with representatives of the national level actors, HELCOM, and the EU. Interviewees represent the domains of politics and administration, national governments, and ministries, as well as national and EU agencies. Also other stakeholders, including representatives of the research communities and interest groups such as farmers’ unions and NGOs, were interviewed in the project. The interviews were thematic and semistructured, implying that questions varied according to the field of expertise of the interviewee, and the answers were of free format, conversational style. The interviews were recorded and transcribed. The number of interviews totaled 171, varying between 10 (Russia) and 33 (EU) (see Table 1).

**Table 1:** Background of interviewed persons in the PROBALT project

	Academia	NGO	Regional authority	National authority	Local authority	Other	TOTAL
Baltic states	9	3	–	10	–	–	22
Germany	4	6	7	5	–	4	26
Finland	18	1	1	10	2	4	36
Poland	8	1	5	2	5	10	31
Sweden	4	3	–	5	1	–	13
Russia	2	4	3	–	1	–	10
European Union	4	8	4	7	–	10	33
Total	49	26	20	39	9	28	171

The interviews and other data were analyzed by applying the empirical methods of qualitative content analysis (Roberts 1997). In general, the analysis proceeded by first identifying the key elements of each country’s national Baltic Sea eutrophication prevention endeavors and the most important mechanisms to support them. Then, the interviews and relevant documents were analyzed with the focus on structural and institutional weaknesses of the present governing system of the Baltic Sea concerning the problem of eutrophication. The challenges identified are presented as four summarizing and interlinked categories.

The approach to studying governance applied in this article is focused on explaining the polity and policy dimensions of governance rather than that of politics (see Pahl-Wostl 2009). That is, instead of explaining the way of policy-making and political processes, we concentrate on analyzing the institutional structures that are in the core of the governing system: the main institutions, policies and actors at each administrative level, and the main governance challenges met at these levels.

## The Governing System of the Mitigation of Eutrophication in the Baltic Sea

### Overview of Governance at the Levels of the EU and HELCOM

Following the enlargement in 2004, the European Union comprises all the Baltic Sea riparian countries except Russia, and a number of noncoastal states that contribute to nutrient input via air pollution (e.g., Great Britain, the Netherlands and Belgium). The EU has developed into an important institutional and legislative power in the protection of the Baltic Sea (see Schumacher 2012). As described by Schumacher (2011a), the first EU regulations aiming to reduce nutrient input into water bodies, the Urban Wastewater Treatment Directive (UWWTD) and the Nitrates Directive (ND), were adopted in 1991. These directives prescribe different standards for sewage treatment and farming practices, respectively. Another instrument for nutrient input reduction is the agri-environmental program of the Common Agricultural Policy (CAP), through which compensation can be paid to farmers who carry out certain water protection measures. Since 2001, airborne emissions of, e.g., nitrogen oxides and ammonia have been addressed in the National Emission Ceilings Directive (NECD). More comprehensive instruments include the Water Framework Directive (WFD), adopted in 2000, and the Marine Strategy Framework Directive (MSFD), adopted in 2008. The main objective of the former is to reach good ecological status of European surface waters and groundwater by 2015, while the latter aims to achieve or maintain such a status in the marine environment by 2020. In order to implement these directives, the member states were invited to develop national water and marine protection plans through which the targets in their territorial waters could be met. By introducing these regulative tools, the EU has taken a stronghold over eutrophication-relevant policy developments in its member states (Schumacher 2011a). More to the point, the EU showed increased interest toward the Baltic Sea region by adopting the first EU macroregional strategy, the Strategy for the Baltic Sea Region (EUSBSR) in 2009. The overall aim of the strategy is to save the sea from environmental degradation, connect the region, and increase prosperity through a number of actions and projects addressing the most relevant challenges and opportunities in the region, including eutrophication.

The HELCOM, in turn, covers the whole region: all coastal countries are contracting parties to the Helsinki Convention. Also other relevant governments (e.g., Belarus and Ukraine) are invited to participate or may apply for an observer status (interview with HELCOM expert, 2010). Over 130 “hot spots,” mostly related to inadequate wastewater treatment, were listed in a HELCOM Joint Comprehensive Environmental Action Program (JCP) in 1992, and many of them were eliminated during the following decade. Currently, the main tool of HELCOM is the Baltic Sea Action Plan (BSAP), agreed in 2007 by the member states to restore the good ecological status of the Baltic Sea by 2021 (HELCOM 2007). The BSAP introduces provisional, countrywise nutrient input reductions targets, which are based on the current nutrient input and the previous reduction efforts. The implementation of the BSAP is carried out through national implementation programs. In recent years, cooperation between the EU and HELCOM regarding the Baltic Sea protection has significantly increased (HELCOM expert, pers.comm. 2010). For example, the national marine strategies of the EU MSFD, as well as the environmental segment of the EU Strategy for the Baltic Sea Region, are based on the regionally agreed environmental goals and objectives (i.e., the HELCOM Baltic Sea Action Plan). In addition, synergies in the implementation processes of the three have been utilized across the region (e.g., Pihlajamäki et al. 2013).

### Overview of the National Governance

The Baltic Sea coastal countries have their own national water and marine policies and legislations that regulate the Baltic Sea related issues in their territories. The national Baltic Sea protection efforts vary in respect of the ambition, the capacity, and experience of environmental administration, funding, and continuity (Jokela 2011; Dmochowska and Szaniawska 2011; Nechiporuk et al. 2011; Pihlajamäki 2011; Schönach 2011; Schumacher 2011b). Some of the countries have aggregated environmental protection strategies that include the Baltic Sea protection policies as one issue among other topics (Estonia, Latvia, Sweden) and others have designed documents targeting especially the Baltic Sea/marine protection issues (Russia, Lithuania, Finland, Poland, Germany) (Pihlajamäki and Tynkkynen 2011b). These strategies include the goals expressed in the EU regulation and, to a varying extent, those of the HELCOM BSAP. National Implementation Programmes of the BSAP vary between the coastal countries remarkably (HELCOM expert, pers.comm. 2010; see HELCOM 2013a).

To characterize the Baltic Sea coastal countries in terms of the protection of the Baltic Sea, Finland and Sweden have often been referred to as “forerunners,” whereas the former state socialist countries are considered “laggards” (Darst 2001). The huge but slowly shrinking gap in the living standards between Eastern and Western shores of the Baltic Sea has materialized in significant differences with regard to the implementation of HELCOM’s recommendations (HELCOM 2003). Moreover, differences in socioeconomic, political, and administrative systems crystallize in societal activities such as cultures of public participation, these in turn conditioning the diffusion of new policy practices especially in Russia. The EU membership of the three Baltic States and Poland—the implementation of environmental *acquis commuanutaire* 3—has naturally brought environmental conditions of these countries closer to those of the Nordic countries and Germany. The EU directives are transposed into the national legislation of each member state. The freedom of the member states in terms of directives’ contents is limited, but national legislations can provide stricter requirements than the directive. For example, in Germany, the urban wastewater purification rates are higher than required by the UWWTD or even by the BSAP (Schumacher 2011b). Moreover, the directives do not explicitly define governance structures and processes; hence, countrywise solutions vary in the role of central agencies and the division of responsibilities between authorities (Pihlajamäki and Tynkkynen 2011b; Pihlajamäki et al. 2013).

Because Russia is perhaps the most important single actor in the current context, not least as it is not a member of the EU, we focus in this article on its national position vis-a-vis the Baltic Sea governance. The case study on Russia reveals that Russia’s input into the Baltic Sea protection activities has been inconsistent because of past socioeconomic difficulties and political instabilities, including repeated reorganising of the environmental administration and blurred division of responsibilities between the federal and regional authorities. This has led to a lack of continuity in environmental policies (interview with a regional authority in Russia, 2009). However, as indicated by the interviewees, Russia has expressed a keen interest in promoting HELCOM as the main actor in environmental protection of the Baltic Sea; especially after the eastern enlargement of the EU to reduce the dominance of the EU in the Baltic Sea region. In a broad sense, Russia and the EU differ in their approaches toward the strategic comprehension of the environmental policy in the Baltic Sea. In spite of the diversity of declared goals of the “National Program of Measures to Improve the Health and Rehabilitation of the Baltic Sea ecosystem” (2012–2020), the Baltic Sea protection appears to be for Russian actors, the first and foremost, a purely technocratic problem, which can be solved by reconstructing wastewater treatment facilities in St. Petersburg, Kaliningrad, and Leningrad oblast regions (interview with a regional authority, Russia 2009; see also Nechiporuk et al. 2011).

## Main Challenges of the Governance of the Eutrophication Mitigation in the Baltic Sea Region

Drawing on the analysis of interviews and other data conducted within the PROBALT project, we identified four sets of challenges as the most serious ones hampering the effective governance of the eutrophication mitigation in the Baltic Sea. In the following, we explicate these challenges and provide some suggestions on how to overcome them.

### The European Union Versus HELCOM

Our analysis reveals that the governing system of the Baltic Sea eutrophication mitigation is the first and foremost hampered by the absence of a supranational regulatory body, or a “primus motor” that would cover all counties in the catchment of the sea as well as other relevant actors. Currently, the two candidates for such a role are the EU and HELCOM. The EU directives play a decisive role in addressing the Baltic Sea eutrophication, but besides not including Russia, these directives unfortunately often fail to consider the unique characteristics of the Baltic Sea. The directives, for example, the UWWTD, are generally less strict than the HELCOM recommendations, and therefore, too lax to effectively combat eutrophication (HELCOM 2006). More recent directives, such as the MSFD and WFD, are more promising in this respect, as their implementation plans are drawn separately to meet the needs of specific marine and water areas. These directives allow, however, the member states under certain conditions to extend the target year to 2027 (WFD) and exceptions in reaching the target (MSFD). This produces an implementation delay that hampers the protection of the Baltic Sea (Schumacher 2011a). Furthermore, agreeing on a more stringent Baltic Sea protection plan within the framework of the EU is difficult if not impossible, as many of the member states, such as the Baltic states (Jokela 2011) and Poland (Dmochowska and Szaniawska 2011), are struggling to fulfill even the currently existing requirements. The EU Common Agricultural Policy (CAP) is also problematic in many ways. Although the EU agri-environmental payment scheme offers financial support to complete water protection projects, due to its inflexibility and long-term commitment requirements as well as laborious bureaucratic paper work related to it, some farmers are reluctant to apply for the same (e.g., interview with a scientist, Sweden 2011). For instance, in Sweden, funds are not being utilized in full (interview with a scientist, Sweden 2011).

Despite these challenges, our analysis indicates that the implementation of the EU directives is a top priority over the HELCOM recommendations in all EU member states (Pihlajamäki and Tynkkynen 2011b). This is the first and foremost point due to the existing enforcement power of the EU, i.e., the member states face significant sanctions if they fail to implement the various directives. The position of the coastal states toward HELCOM, in turn, is more multifaceted (e.g., interviews with two civil servants and one NGO representative in Sweden 2011; Dmochowska and Szaniawska 2011; Jokela 2011). HELCOM recommendations have been enforced to a varying extent in national policies and legislation of the coastal states. The main deficiency of HELCOM is that, contrary to the EU framework directives, sanctions for noncommitment of the HELCOM recommendations do not exist (HELCOM expert, pers.comm. 2010; VanDeveer 2011). This generates lack of commitment, and hence, recommendations put forth by HELCOM do not materialize effectively enough in national regulations, let alone in implementation. The BSAP as the main tool of HELCOM to manage eutrophication has been accused by NGOs for not including those ambitious actions originally included in the proposals of the plan, for not treating the countries equally, and for forgetting to cover socioeconomic aspects. More to the point, HELCOM is criticized for concentrating mainly on scientific activities and accumulating data instead of supporting the concrete implementation of the proposed measures (interviews with two NGO representatives in Sweden 2011; interview with a scientist in Finland, 2009).

On a more positive note, the greatest assets of HELCOM include the participation of all nine coastal countries, the possibility to address the whole catchment area, and its long history and distinguished role as a forum for the macroregional Baltic Sea protection. HELCOM has also resonance in Russia, where, as a non-EU member, HELCOM is the main transnational governing body for the environment of the Baltic Sea (e.g., interview with a scientist in Russia, 2009). As a major polluter and a geopolitical actor, Russia’s interests need careful consideration. This implies that the position of the EU as the “primus motor” of Baltic Sea environmental protection is not self-evident, even if more stringent EU regulation that takes specific conditions of the Baltic Sea and the eutrophication problem into account is allowed to be in place.

### Socioeconomic Differences and the Lack of Environmental Awareness

The historically rooted differences of the countries’ socioeconomic situations and capacities are among the key challenges for effective governance of the Baltic Sea environment in general and eutrophication mitigation in particular. We argue that existing policies do not take these differences into account in an adequate manner. Above all, this applies to the HELCOM BSAP, in which the highest nutrient reduction requirements are set for developing economies in the region, namely Russia, Poland, Latvia, and Lithuania, leaving Finland and Estonia with the lightest burden. Thus, the minimized cost for implementation of the plan, calculated up to €4.7 million annually (Wulff et al. 2014), is very unevenly distributed compared with the GDP or other indicators of economic performance. This is regarded as an unfair division of burden and lessens the incentive for implementation, for example, in Poland (Dmochowska and Szaniawska 2011).

Another example of how socioeconomic differences affect the governance of eutrophication mitigation is provided by the agricultural sector. Before the 2004 EU enlargement, the intensity of agricultural production and the consequent use of fertilizers in, for example, Poland were moderate mainly due to poor economic conditions. However, the EU CAP has promoted intensified agricultural production throughout the region and is expected to result in substantial growth of the agricultural sector in Poland and subsequently increased nutrient leaching (Dmochowska and Szaniawska 2011). At the same time, the implementation of environmental measures to reduce nutrient run-off through the national agri-environmental programs (as part of the EU CAP) depend on the willingness and possibility of the state to provide co-funding from their national budgets (e.g., interview with an environmental authority, Finland 2011). In Poland, it remains yet to be seen how environmental protection will be guaranteed in the intensified agricultural production (Dmochowska and Szaniawska 2011).

With regard to the awareness concerning the problem of eutrophication, our analysis supports the findings of Flash Eurobarometer on Water (2009) and Ahtiainen et al. (2012), which suggest that eutrophication is perceived as the most severe environmental problem of the Baltic Sea in Finland and Sweden, but not in Germany, Poland, the Baltic States and Russia. This can be explained by geographic, geopolitical, and historic-cultural reasons: Finland and Sweden have the longest Baltic Sea coastlines, economic activities concentrated along the coast and long traditions of Baltic Sea related livelihoods, especially recreation. In Russia, Germany and Poland, the geographic distances of the capital cities from the Baltic Sea downplay the national significance of the problems of the Baltic Sea. This is reflected in that public knowledge concerning the Baltic Sea eutrophication is somewhat poor and/or scientifically contested, and media attention is scarce (Dmochowska and Szaniawska 2011; Jokela 2011; Nechiporuk et al. 2011; Schumacher 2011b). In the former socialist countries, open access to information and public deliberation is a new aspect; therefore, NGOs and other actors have only relatively recently started to participate in the environmental protection of the Baltic Sea (Jokela 2011). At the level of the EU, the good environmental reputation of the northern European countries leads to the false impression of a good environmental status of the Baltic Sea (Schumacher 2011a).

Weak or pending environmental awareness poses a real threat to the target of achieving a good ecological status of the Baltic Sea. Growingly important instruments of environmental policy-making such as public pressure, everyday activism and environmentally friendly business culture all benefit from a higher environmental awareness. Truly effective policies can be planned only with the help of those actors whose actions are critical with regard to the problem (Haila 2008). Various stakeholders are also in the key position when searching for cost-efficient measures to decrease nutrient leakage. Enhancing cost-efficiency is closely related to the question of prioritization of policies. This, however, necessitates awareness on the problem and personal interest: the implementation of protective measures often means costs to be carried even by individual citizens, such as farmers or house owners. If not provided with additional funding and especially when combined with a lack of awareness, other expenditures may appear more pressing.

### Inadequate Spatial and Temporal Specification of Policies

The existing governing system does not efficiently reach out to all sources of nutrient leaching along the coastal zones and in river catchments. This concerns agriculture in particular, as the magnitude of nutrient leaching varies significantly among the cultivated areas (e.g., interviews with one scientist, one local politician, one civil servant at MoA, and one NGO representative, Sweden 2011; interview with two scientists, Finland 2009). This implies that instead of implementing agri-environmental measures equally across the region, the measures should be focused where the benefit (i.e., the amount of nutrient reduction) is the greatest. In general, this refers to cost-effective practices that introduce spatially specific environmental measures. Temporal specification, in turn, implies the concentration of nutrient reduction efforts to sources from which the greatest benefit (nutrient reduction) can be achieved the quickest (interview with a scientist in Finland, 2009). Specification necessitates also a bottom-up approach focusing on specific situations and differentiating management practices with the strong involvement of relevant stakeholders (interview with one scientist and one local politician, Sweden, 2011; see also Haila 2008).

Based on our analysis, the existing policy instruments, especially those included in the CAP, do not facilitate effectively enough such spatial differentiation of policy instruments and forms of implementation and have thus led to inaccurate allocation of responsibilities and waste of funds targeted to protection. Instead of focusing the measures to those areas where the potential benefit is the greatest, the agri-environmental measures of the CAP are implemented equally across the region. Participation in the national agri-environmental programs is voluntary for the farmers and includes a certain degree of freedom in the selection of measures, which can also lead to an ineffective allocation of measures in respect of eutrophication sensitive areas, or in the worst case scenario, a complete lack of them (e.g., interview with one scientist, Finland 2009; interview with environmental authority, Finland 2011). More to the point, not only the EU regulation but even some national funding systems for water protection, e.g., in Sweden, do not take spatial differences into account carefully enough, and hence protective measures are not carried out cost-effectively (e.g., interviews of two civil servants at the Swedish Environmental Protection Agency, two NGO representatives and one scientist, Sweden 2011; see also Lundqvist 2004). To some degree, spatial and temporal inflexibility of impacts of measures is caused by inadequate follow-up and evaluation and malfunctioning feedback mechanisms (e.g., interviews with one local politician, one scientist and two civil servants at the Swedish Environmental Protection Agency, Sweden 2011). The best possible results are not reached if action cannot be readjusted when the follow-up information identifies improvement needs.

To advance temporal specification of policies, intermediate steps toward final goals that are connected to technological possibilities and would thus facilitate implementation of policies need to be defined (interview with a university scientist, Finland 2009). Such a temporal dimension is built in NEFCO’s (Nordic Environmental Finance Corporation) suggestion to develop a nutrient trading system in the Baltic Sea (NEFCO 2008). Starting nutrient trading between point sources (e.g., urban wastewater treatment plants) would bring reductions in nutrient loads quickly in contrast to the inevitably slow progress achievable in agriculture (Ministry of Agriculture and Forestry of Finland 2010; Lankoski and Ollikainen 2011). Reduction of nutrient loads from urban wastewater treatment plants by using a trading mechanism would constitute roughly 70 % of the reduction targets set out in the BSAP (Hautakangas and Ollikainen 2011). This reduction is large and would, based on the consequent changes in water quality and algal blooms, provide an opportunity to reconsider and fine-tune the BSAP’s targets. Actually, this is what the most recent 2013 HELCOM Ministerial Declaration urges: it is agreed in the Declaration that further upgrading of waste water treatment to fully implement the relevant HELCOM Recommendation will be prioritized (see HELCOM 2013b).

### Insufficient Integration of Different Policy Sectors

Various activities throughout the catchment area (and beyond) either directly or indirectly affect the state of the sea. Therefore, the state of the Baltic Sea cannot be improved by focusing exclusively on marine and water protection, if all societal activities are not closely linked to protective activities. Some of the EU policies are often even contradictory, e.g., agricultural and environmental policies, suffering from the lack of proper integration (Kern 2011; Schumacher 2011a). In the EU, agricultural decision-making has taken place within a rather isolated policy network for many decades, and in many national cases strong farming lobbyists are against more stringent environmental policies while fearing for the loss of competitiveness (interviews of two NGO representatives, Sweden 2011). The CAP is not in synergy with the WFD and MSFD, instead the CAP gives counterproductive incentives to nutrient input reduction by encouraging farmers to increase both area under cultivation and intensity of production (e.g., interview with a scientist, Finland 2009; see also Lankoski and Ollikainen 2011). As a result environmental impacts of agricultural policy actually undermine the achievements of agri-environmental policy regarding water protection. Furthermore, the agri-environmental programs can be targeted toward a multitude of different environmental goals and there is no legal obligation to prioritize water protection (Guttenstein 2007). In fact, the Commission has even requested Finland not to increase the share of water protection related measures in its national program (interview with a scientist and a former representative of farmers union in Finland, 2011). Strong policy fragmentation calls for an exhaustive reform, which unfortunately the new CAP does not seem to be bring about.

A better integration of policy sectors presupposes the explication of interrelations between different policy areas, taking the specific spatial, temporal and administrative scales of the activities that are causing the problem as the starting point for policy integration. In addition, trade-offs and synergies between different policy, environmental and agricultural sectors (also sectors like transportation, energy, housing, fisheries) should be more systematically taken into consideration at every governance level. Positive effects—double benefits—could be expected both in regard to other environmental objectives (e.g., climate protection and biodiversity) and in terms of socioeconomic interests (e.g., improved drinking-water quality, cost savings by increasing fertilizer efficiency and improved conditions for the tourism and fisheries sectors) (interview with one local politician and one scientist, Sweden, 2011; Schumacher 2011a).

## Concluding Remarks

The examination of the governing system of the Baltic Sea eutrophication mitigation reveals that the main challenges for more effective governance are rooted in the boundaries of different levels of the governing system and their interactions. Furthermore, historically rooted differences in socioeconomic circumstances, between the Nordic countries and Germany, and the former socialist states, Russia in particular (see Jahn and Kuitto 2008; Kern, 2011), pose yet another key challenge.

The existing governing system is a rather state-centric one (Kern 2011). The state-centricity is an imperative that cannot be forgotten, because there is no supranational governing body that would transcend the sovereignty of all countries in the catchment area. As brought up by some of the experts interviewed in the PROBALT project, one option to solve this problem would be a binding regional agreement that would contrary to the existing Helsinki Convention be fair, cost-effective and include mechanisms of sanctions for noncompliance. However, such an agreement may not be plausible given that the Helsinki Convention is already in place. At minimum, the governing system is in need of enhanced interplay between the existing institutions and policies (see Oberthür and Schram Stokke 2011). The EU Strategy for the Baltic Sea Region provides a good start, especially vis-à-vis the interplay between the EU MSFD and WFD and the HELCOM Baltic Sea Action Plan. However, action driven strongly by the EU excludes Russia. Furthermore, the dominant role of the EU in the Baltic Sea protection is not greeted by Russia, anxious to rebuild its former superpower status. Therefore, the HELCOM is more likely to continue to be an important actor also in the future, and its policies should be revised accordingly.

Furthermore, stakeholder engagement is a major consideration that should be taken into the core of any development not only to gain political legitimacy but also for the purposes of spatial and temporal specification. Without connecting with “on the ground” constituencies, the governance system is unable to effectively reach out to all sources of eutrophication in the catchment area and to correct scale discrepancies between the problem and policy instruments (Haila 2008). Improved stakeholder participation would encourage a change from the use of direct top-down regulations to the introduction of incentive-based flexible instruments and individual voluntary efforts to protect the sea (see Hammer et al. 2011). But increasing public participation is not an easy task and cannot be carried out artificially on top of existing procedures. At the core of enhancing broad stakeholder participation and legitimation of policies is wide public acceptance and desirability of the goals. This necessitates awareness of the problem at all levels. Awareness can be raised by increasing the publicity and media attention concerning the problem of eutrophication. Events like the Baltic Sea Action Summits are very important in this respect; also environmental education plays a key role especially in the long term.

In this article, we did not analyze the role of subnational actors, although especially the EU legislation is often implemented at local and regional levels (Kern 2011). More to the point, nongovernmental actors for the governance of the mitigation of the Baltic Sea eutrophication were left out of the scope of this article. This was because the focus of the PROBALT project was on the formal governing system. It should be noted, however, that subnational actors as well as nongovernmental actors have a growing role in policy making and also in the implementation of already negotiated policies. Following from this notion, a number of conclusions with regard to future research needs can be drawn. From the viewpoint of policy analysis, it would be important to focus sustained inquiry not only on the ways in which spatial and temporal specification of policies could be carried out, but also on how policy integration could be organized across policy sectors and levels and actor groups in a way that encourages participation and the inclusion of bottom-up perspectives in policy-making. Last but not least, to guarantee the requirement of visibility of the problem and to increase knowledge on it, all sort of research should pay special attention to the communication of results to the wider public also in the future.

## Footnotes

1 The Baltic Sea encompasses nine coastal countries: Denmark, Germany, Poland, Lithuania, Latvia, Estonia, Russia, Finland, and Sweden. The other countries in the catchment area are Czech Republic, Slovak Republic, Ukraine, Belarus, and Norway.

2 Due to *a force majeure*, a case study on Denmark was unfortunately not conducted.

3 The entire body of European legislation, comprising all the treaties, regulations, and directives adopted by the European Union.

## References

[CR1] Ahtiainen, H., L. Hasselström, J. Artell, D. Angeli, M. Czajkowski, J. Meyerhoff, M. Alemu, K. Dahlbo, et al. 2012. Benefits of meeting the Baltic Sea nutrient reduction targets—Combining ecological modeling and contingent valuation in the nine littoral countries. *MTT Discussion Papers 1*. http://www.mtt.fi/dp/DP2012_1.pdf.

[CR2] Darst, R.G. 2001. *Smokestack diplomacy. Cooperation and conflict in East—West environmental politics*. Cambridge: The MIT Press.

[CR3] Dmochowska B, Szaniawska A, Pihlajamäki M, Tynkkynen N (2011). Poland—Looking for a higher environmental awareness. Governing the blue-green Baltic Sea.

[CR4] Flash Eurobarometer on Water. 2009. No 261. Available from http://ec.europa.eu/public_opinion/flash/fl_261_en.pdf.

[CR5] Gänzle S (2011). Introduction: Transnational governance and policy-making in the Baltic Sea region. Journal of Baltic Studies.

[CR6] Guttenstein E (2007). The potential of the European agricultural fund for rural development (EAFRD) to address diffuse pollution from agriculture and consequent eutrophication of the Baltic marine environment.

[CR7] Haila, Y. 2008. Unity versus Disunity of Environmental Governance in the Baltic Sea Region. In *Governing a common sea. Environmental policies in the Baltic Sea Region*, ed. M. Joas, D. Jahn and K. Kern, 193–212. London: Earthscan.

[CR8] Hammer M, Balfors B, Mörtberg U, Petersson M, Quin A (2011). Governance of water structures in the phase of change: A case study of implementation of the EU Water Framework Directive in Sweden. AMBIO.

[CR9] Hautakangas S, Ollikainen M, Pihlajamäki M, Tynkkynen N (2011). Making the Baltic Sea Action Plan workable: A nutrient trading scheme. Governing the blue-green Baltic Sea.

[CR10] HELCOM. 2003. The review of more specific targets to reach the goal set up in the 1988/1998 Ministerial Declarations regarding the nutrients. Baltic Sea Environmental Proceedings No. 89.

[CR11] HELCOM. 2006. *Eutrophication in the Baltic Sea. Draft HELCOM Thematic Assessment in 2006*. Helsinki: HELCOM. Retrieved from http://helcom.navigo.fi/stc/files/BSAP/FINAL%20Eutrophication.pdf.

[CR12] HELCOM. 2007. *HELCOM Baltic Sea Action Plan.* Helcom Ministerial Meeting Krakow, Poland, 15 November 2007. Retrieved from http://www.helcom.fi/stc/files/BSAP/BSAP_Final.pdf.

[CR13] HELCOM. 2008. *Convention on the Protection of the Marine Environment of the Baltic Sea Area, 1992 (Helsinki Convention)*. Retrieved from http://www.helcom.fi/stc/files/Convention/Conv1108.pdf.

[CR14] HELCOM. 2009. Eutrophication in the Baltic Sea. An Integrated Thematic Assessment of the Effects of Nutrient Enrichment in the Baltic Sea region. Baltic Sea Environment Proceedings No. 115B.

[CR15] HELCOM. 2013a. National Implementation Programmes (NIP). Retrieved 2 August, 2013, from http://www.helcom.fi/BSAP/Implementation/en_GB/Implementation/.

[CR16] HELCOM. 2013b. *The 2013 HELCOM Ministerial Declaration. Fourth draft of the Copenhagen Ministerial Declaration. 2/7/Rev.1.* Helsinki: Helsinki Commission. Retrieved 24 September, 2013, from http://meeting.helcom.fi/c/document_library/get_file?p_l_id=18975&folderId=2351178&name=DLFE-54671.pdf.

[CR43] Hjorth R (1996). Baltic environmental cooperation: A regime in transition.

[CR17] Jahn, D., and K. Kuitto. 2008. Environmental pollution and economic performance in the Baltic Sea region. In *Governing a common sea. Environmental policies in the Baltic Sea Region.* ed. M. Joas, D. Jahn and K. Kern, 83–111. London: Earthscan.

[CR18] Joas, M., Jahn, D., and K. Kern. 2008. Governance in the Baltic Sea region: Balancing states, cities and people. In *Governing a common sea. Environmental policies in the Baltic Sea region,* ed. M. Joas, D. Jahn, and K. Kern, 3–15. London: Earthscan.

[CR19] Jokela M, Pihlajamäki M, Tynkkynen N (2011). The Baltic States—At a crossroads of different environmental development paths. Governing the blue-green Baltic Sea.

[CR21] Kern K (2011). Governance for sustainable development in the Baltic Sea region. Journal of Baltic Studies.

[CR22] Lankoski, J., and Ollikainen, M. 2011. Counterfactual approach for assessing agri-environmental policy: Theory with an application to Finnish water protection policy. University of Helsinki, Department of Economics and Management. Discussion Papers No. 56. Helsinki: University of Helsinki.

[CR23] Lundberg C (2013). Eutrophication, risk management and sustainability. The perceptions of different stakeholders in the northern Baltic Sea. Marine Pollution Bulletin.

[CR24] Lundqvist L (2004). Sweden and ecological governance: Straddling the fence.

[CR25] Ministry of Agriculture and Forestry of Finland. 2010. Follow-up Study on the Impacts of Agri-Enviroment Measures (MYTVAS 3)—Mid-term Report. Retrieved from http://www.mmm.fi/attachments/mmm/julkaisut/julkaisusarja/newfolder/5pe9soaAU/Mytvas_netti.pdf.

[CR26] Nechiporuk D, Nozhenko M, Belokurova E, Pihlajamäki M, Tynkkynen N (2011). Russia—a special actor in Baltic Sea environmental governance. Governing the blue-green Baltic Sea.

[CR27] NEFCO (Nordic Environment Finance Corporation). 2008. Framework for a nutrient quota and credit’s trading system for the contracting parties of HELCOM to reduce eutrophication of the Baltic Sea. JR-080229-P5320-005, NEFCO, Helsinki.

[CR28] Oberthür S, Schram Stokke O (2011). Managing institutional complexity. Regime Interplay and Global Environmental Change.

[CR29] Pahl-Wostl C (2009). A conceptual framework for analysing adaptive capacity and multi-level learning processes in resource governance regimes. Global Environmental Change.

[CR30] Pihlajamäki M, Pihlajamäki M, Tynkkynen N (2011). Finland—No easy solutions left. Governing the blue-green Baltic Sea.

[CR32] Pihlajamäki M, Tynkkynen N (2011). The challenge of bridging science and policy in the Baltic Sea eutrophication governance in Finland: The perspective of science. AMBIO.

[CR33] Pihlajamäki M, Tynkkynen N (2011). Governing the blue-green Baltic Sea.

[CR31] Pihlajamäki, M., R. Varjopuro, M. Nekoro, M. Valman, E. Roth, I. Psuty, E. Andrulewicz, W. Pelczarski, et al. 2013. Marine Strategies for the Baltic Sea—First steps in the implementation of MSFD in Denmark, Poland, Finland and Sweden. Deliverable 7.3 of EU FP7 KNOWSEAS Project (Knowledge-based Sustainable Management for Europe’s Seas).

[CR34] Räsänen T, Laakkonen S, Joas M, Jahn D, Kern K (2008). Institutionalization of an International Environmental Policy Regime: The Helsinki Convention, Finland and the Cold War. Governing a common sea: Environmental policies in the Baltic Sea region.

[CR35] Roberts CW (1997). Text analysis for the social sciences: Methods for drawing inferences from texts and transcripts.

[CR36] Schönach P, Pihlajamäki M, Tynkkynen N (2011). Sweden—A pioneer with implementation inefficiencies. Governing the blue-green Baltic Sea.

[CR37] Schumacher T, Pihlajamäki M, Tynkkynen N (2011). The capacity of the EU to address marine eutrophication. Governing the blue-green Baltic Sea.

[CR38] Schumacher T, Pihlajamäki M, Tynkkynen N (2011). Germany—No priority of Baltic Sea protection. Governing the blue-green Baltic Sea.

[CR39] Schumacher T (2012). Great potential but little impact: The European Union’s protection policies for the Baltic Sea. Baltic Journal of Political Science.

[CR40] VanDeveer S (2011). Networked Baltic environmental cooperation. Journal of Baltic Studies.

[CR41] Wulff F, Savchuk O, Sokolov A, Humborg C, Mörth C-M (2007). Management options and effects on a marine ecosystem: Assessing the future of the Baltic. AMBIO.

[CR42] Wulff, F., C. Humborg, H.E. Andersen, G. Blicher-Mathiesen, M. Czajkowski, K. Elofsson, A. Fonnesbech-Wulff, B. Hasler, et al. 2014. Reduction of Baltic Sea nutrient inputs and allocation of abatement costs within the Baltic Sea catchment. *AMBIO*. doi:10.1007/s13280-013-0484-5. 10.1007/s13280-013-0484-5 PMC3888655 24414801

